# Nurses’ knowledge, behaviour and compliance concerning hand hygiene in nursing homes: a cross-sectional mixed-methods study

**DOI:** 10.1186/s12913-019-4347-z

**Published:** 2019-08-05

**Authors:** Judith Hammerschmidt, Tanja Manser

**Affiliations:** 1 Institute for Patient Safety, University Hospital Bonn, Venusberg Campus 1, 53127 Bonn, Germany; 2 University of Applied Sciences and Arts Northwestern Switzerland, FHNW School of Applied Psychology, Olten, Switzerland

**Keywords:** Infection prevention, Hand hygiene, Nursing homes, Nurses role, Nursing manager, Nursing, Patient safety

## Abstract

**Background:**

Effective hand hygiene is one of the most important measures for protecting nursing home residents from nosocomial infections. Infections with multi-resistant bacteria’s, associated with healthcare, is a known problem. The nursing home setting differs from other healthcare environments in individual and organisational factors such as knowledge, behaviour, and attitude to improve hand hygiene and it is therefore difficult to research the influential factors to improve hand hygiene. Studies have shown that increasing knowledge, behaviour and attitudes could enhance hand hygiene compliance in nursing homes. Therefore, it may be important to examine individual and organisational factors that foster improvement of these factors in hand hygiene. We aim to explore these influences of individual and organisational factors of hand hygiene in nursing home staff, with a particular focus on the function of role modelling by nursing managers.

**Methods:**

We conducted a mixed-methods study surveying 165 nurses and interviewing 27 nursing managers from nursing homes in Germany.

**Results:**

Most nurses and nursing managers held the knowledge of effective hand hygiene procedures. Hygiene standards and equipment were all generally available but compliance to standards also depended upon availability in the immediate work area and role modelling. Despite a general awareness of the impact of leadership on staff behaviour, not all nursing managers fully appreciated the impact of their own consistent role modelling regarding hand hygiene behaviours.

**Conclusion:**

These results suggest that improving hand hygiene should focus on strategies that facilitate the provision of hand disinfectant materials in the immediate work area of nurses. In addition, nursing managers should be made aware of the impact of their role model function and they should implement this in daily practice.

## Background

Healthcare-associated infections are a major cause of morbidity and mortality in nursing homes [1, 2]. In 2015 there were 426.277 cases of infections with multi-resistant bacteria associated with health care registered in Europe [3]. The most effective single measure for infection prevention in various health care settings, including nursing homes is (antiseptic) hand rubbing [4–6].

This term refers to “applying an antiseptic handrub to reduce or inhibit the growth of microorganisms without the need for an exogenous source of water and requiring no rinsing or drying with towels or other devices” [7]. The 5 moments of hand hygiene define care situations that should always lead to a hand rub [7]. Previous research has pointed to individual knowledge deficits influencing safe hand hygiene practices in nursing homes such as correct duration of hand washing and deficits in hand rub recommendations [8–10]. It was also shown that the incidences of serious infection could be reduced after the introduction of a multifaceted hand hygiene program to improve hand hygiene adherence and compliance in nursing homes [11, 12]. However, it has been pointed out that the application of hospital infection control guidelines to nursing homes is often unrealistic in terms of system differences and different available resources for infection prevention [13]. For example, the absence of a sink was found to be a major hindrance to hand hygiene in the nursing home setting [14]. Individual factors of nurses such as knowledge of the 5 moments of hand hygiene, behaviours including not wearing hand and arm jewellery while nursing, and applying their learnings from the latest hygiene training, to improve compliance of hand hygiene measures, are important prerequisites for infection prevention. Yeung et al. [11] showed that hygiene programmes and education could effectively increase adherence to hand rubbing and reduce the incidence of serious infections in nursing homes. Thus, apart from these individual factors, effective hand hygiene also requires adequate organisational factors including availability of hand rub, stock of protective clothing, and strong local efforts from the nursing management such as role modelling. Role modelling is defined by Merton as; a person who sets a positive example and is worthy of imitation [15]. Huis et al. have shown that hand hygiene was performed more frequently when group members with a higher hierarchical position disinfected their hands [16]. Schneider et al. found that adherence of junior practitioners improved under the supervision of adherent role models [17]. Furthermore, Lankford et al. pointed out that healthcare workers in the presence of a senior who is not washing his hands are also less likely to wash their hands [18]. In contrast to other care settings, improvements in nursing homes are often compromised by the prevailing goal conflict between preserving a homelike environment and social care on the one hand, and the adoption and control of infection prevention measures on the other [13, 19, 20]. In times of demographic change and the post-antibiotic era, the challenges to organisational and individual framework conditions in nursing homes are shifting. Residents’ expectations of the time they spend in nursing homes have changed in terms of quality of life, active participation and protection against multi-resistant pathogens [21, 22]. Generally, the interventional approaches to hand hygiene in nursing homes do not seem to differ from those in other care settings. However, in the nursing home setting, change processes towards improved hand hygiene outcomes are often non-transparent [8, 9, 14, 23]. While a large proportion of multidrug-resistant infections in nursing homes could be avoided through appropriate hand hygiene behaviour of nurses, this behaviour is influenced by organisational factors such as hygiene training, availability of resources and improved role modelling of nursing managers [24–28].

This study intends to contribute to an improved understanding of infection prevention with a focus on hand hygiene in nursing homes. We explore the impact of organisational factors on hand hygiene behaviour with a particular focus on role modelling. We combine the perspectives of nurses and nursing managers to do this.

## Methods

### Aim of the study

The aim was to improve understanding of the organisational factors related to compliance with infection prevention management, focussing on hand hygiene in nursing homes. Our research questions considered the perspectives of nurses and nursing managers on their hand hygiene knowledge (*What knowledge do nurses have / How do nursing managers perceive nurses’ knowledge concerning hygiene management and infection prevention?*), their hygiene practices and compliance with hygiene guidelines (*Which hygiene behaviours do nurses report / nursing managers observe in their staff?*) as well as how these behaviours are supported or hindered by organisational aspects and role modelling by nursing managers (*What are the perceptions of nurses and nursing managers of organisational structures and processes supporting hand hygiene? In what way do nurses perceive nursing managers / do nursing managers regard themselves as role models for hand hygiene?*). We applied a mixed-methods approach, collecting survey data on nurses’ knowledge, behaviour, and compliance regarding hand hygiene as well as interview data on nurse managers’ perspectives of organisational influence on infection prevention, to explore multiple perspectives in relation to our research questions. We provide a rich description of the organisational factors that have to be considered when aiming to improve hand hygiene in the nursing home setting.

#### Context of the study

This study was part of a larger cross-interventional project (2012–2015) which aimed to positively influence infection prevention practices, with a focus on hand hygiene in nursing homes for elderly care, through educational and supportive measures for nurses and general practitioners, to improve hygiene practices and rational use of antibiotics. This study reports on the baseline assessment from the nursing perspective.

#### Study setting

A pool of 542 nursing homes was identified. After purposeful sampling i. e. nursing homes caring for older residents each with a mix of care levels ranging from basic support to full nursing provision to meet all aspects of resident care’ and a minimum of 80 residents per nursing home, six institutions were randomly selected and invited to participate in the project. The participating nursing homes cared for 80–130 residents and have four to eight residential areas. In the participating nursing home, there were several managerial and nursing roles: The nursing home managers held the overall responsibility for the whole facility including all employees. They each had a nursing background and additional management qualifications. Nursing managers were responsible for ensuring continuous quality of care and had the responsibility for all nursing staff. In addition to being fully qualified nurses, they had additional training or an academic degree in nursing management. Nursing staff was registered or geriatric nurses. Registered nurses had received three years of training before state examination. This qualifies them to work in acute or long-term care in hospitals, nursing homes and ambulatory care settings without additional training. Geriatric nurses had an additional three years of specialty training with state examination. Geriatric nurses are qualified to work in the care and support of the older people in nursing homes, ambulatory care settings or hospitals with a specialisation of geriatric medicine. They were responsible for the quality and evaluation of the care plan, the practical training of nursing students and treatments such as wound care and the administration of drugs. Nursing aids with one year (or no formal) training follows the nursing care plan while working directly with the residents. All hygiene representatives were registered nurses with additional training in hygiene and infection prevention.

#### Study design

We employed a mixed-methods design with a concurrent triangulation strategy to support our analyses from multiple sources [29]. Quantitative and qualitative data were analysed independently by several researchers. At the triangulation stage, both data sources were combined and given equal weight in the interpretation of data. This approach of integrating findings from the quantitative and qualitative strand of the study at the interpretation stage contributes to a more complete, balanced and insightful portrait of the phenomena under investigation [29, 30].

### Quantitative strand

#### Staff survey

For the PänosInAA study, we developed a survey for nurses in German based on a literature overview that focuses on the perceived knowledge and behaviour of nurses in nursing homes. Content expert members of the research team were involved in its development and cognitive pretesting of the survey items through an iterative process involved five nurses working in older people care. The survey was intended as a tool to collect descriptive data for a series of independent items, not as a questionnaire designed to measure underlying constructs [29, 31]. In line with the research questions, the survey covered the following topics:

- knowledge of hand hygiene (e.g. duration of hand rub);
- perceived behaviours concerning hand hygiene;
- perceived compliance with hygiene standards and integrated hygiene training in practice;
- organisational management of hygiene issues (e.g. communication between nurses and nursing managers, general practitioners);
- organisational factors related to structures and processes hindering or facilitating hand hygiene practices (e.g. access to gloves); and
- perceived role modelling by nursing managers.

The survey comprised 23 main questions, five of which had a total of 34 subcategories. To obtain more detailed information most items could be answered in subcategories and multiple answers were possible. In order to capture data on nurses’ knowledge, we used nominal response categories (i. e. “correct” and “wrong”). For other topics, we used items with a five-point Likert scale with response categories “always”, “often”, “sometimes”, “rarely” and “never”. The survey did not contain any open-ended questions. Divulging socio-demographic data was optional. We calculated the percentages of participants answering “always” or “often”on each item. To explore the differences between the main groups (i. e. Registered nurses, nursing aides/students) we used Fisher’s exact test on all survey data (see Table 2). The full survey is not published and available from the corresponding author on request. However, the relevant items for this study are provided in Table 2.

#### Data collection

During January through March 2013, we conducted a baseline survey with nurses with different levels of training in the participating nursing homes. This was prior to any training intervention relating to the overall project.

### Qualitative strand

#### Data collection

An interview guide, based on our initial literature review Method. In line with the survey for nursing staff it consisted of open-ended questions concerning the following topics:

- contact persons regarding questions concerning infection prevention;
- hygiene topics in handovers;
- possibilities of hand hygiene during care;
- accessible supply of hygienic material;
- compliance to hygiene standards; and
- role modelling with regard to infection prevention.

To identify similarities and differences between particular aspects of phenomena [29] in relation to our research questions, we invited 36 nursing managers from the participating nursing homes for semi-structured interviews in February and March 2013. Acknowledging their managerial experience, individual perspectives and perceived influence on hygiene management and hand hygiene, we explored their multiple perspectives until data saturation was obtained [32, 33].

Each interviewee was informed about the purpose and voluntary nature of the study, data anonymity and security, interviewers’ professional background and role in the project. After obtaining informed consent, interviews were conducted by a team comprising of one lead interviewer and one or two observers with a background in nursing. The duration of the interviews was not fixed. All interviews were audio-recorded and transcribed according to standard linguistic conventions [34].

### Data analysis

#### Quantitative data analysis

During a quality check, surveys with missing value rates ≥80% were excluded from the analysis. Survey results were summarised via descriptive statistics (mean, standard deviation, frequency of each answer (see Table 2). Data management and analysis were conducted using the IBM software SPSS for Windows release version 22 (SPSS, Inc., 2013; Chicago, IL; http://www.spss.com).

#### Qualitative data analysis

All audio-recordings of interviews were anonymised during transcription. Interview transcripts were discussed by a multidisciplinary team consisting of 9 researchers with backgrounds in medicine (2), healthcare management (2), nursing science (2) and psychology (3), in weekly meetings and analysed for emergent themes following an investigator triangulation approach [35]. The themes emerging from this analysis were grouped into six major categories:

- perceptions of nurses’ knowledge concerning hygiene standards;
- perceptions of impact for nurses’ hygiene training;
- nursing managers’ perceptions of nurses’ hand hygiene behaviours;
- nursing managers’ perceptions of nurses’ compliance with hygiene standards;
- nursing managers’ perceptions of organisational factors facilitating or hindering hand hygiene; and
- nursing managers’ reflections on their function as role models.

These themes were then used by the researchers for interview coding (using software MAXQDA version 11; Copyright ©1995–2017, VERBI GmbH). Coding discrepancies were discussed among the researchers and resolved by consensus. In a final step, each transcript was individually summarised to a content analysis following the principles of Bogdan and Biklen [36]. This extract allowed for interpretation at the individual level as well as for comparison between nursing homes.

#### Concurrent triangulation

During concurrent triangulation [29] the relationships, differences, and interactions between the mixed data and the theoretical concept of the study became apparent. During this process, the different perspectives and inputs from the multidisciplinary research team were crucial. Their professional experiences and theoretical backgrounds allowed for a diverse discussion and deep reflection of affirmative and contrasting results.

## Results

### Quantitative strand: staff survey results

The overall response rate was 42% (183 out of 431 surveys). We excluded (*n* = 18; 10%) surveys due to ≥80% missing values. Our final sample was 165.

#### Survey participants

The majority of the sample was female (*n* = 132; 80%) (Table 1). Survey respondents were licensed nurses (*n* = 85; 52%) with an average age of 47 years. The majority of nurses had job tenures of ≤5 years in their institution (*n* = 46; 28%) and worked in day shifts (*n* = 104; 63%).

**Table 1:** Characteristics of survey and interview participants

Survey participants					Interview participants	
Participant characteristics	Frequency (*N* = 165)	Mean	SD +/−	% of sample	Frequency (*N* = 27)	% of sample
Gender	148			90	27	100
Female	132			80	24	89
Male	16			10	3	11
Age	123	47	12	75	27	100
< 29	11			7	2	7
30–39	27			16	2	7
40–49	23			14	8	30
50–59	50			30	9	33
> 60	12			7	6	22
Staff profession	155			94	27	100
Director of Nursing	–			–	6	22
Licensed Nurse / Geriatric Nurse	85			52	15	56
Nursing Aid / Nursing Assistant	65			39	–	–
Nursing Students	5			3	–	–
Hygiene Specialist (nursing background)	–			–	4	15
Hygiene Specialist (other professional backgrounds)	–			–	2	7
Shift	149			90	27	100
Day	104			63	27	100
Night	6			4	–	-
Day and Night	39			24	–	-
Job tenure in the institution (years)	128	10	7	78	27	100
< 5	46			28	4	15
6–10	25			16	4	15
11–15	26			16	8	30
16–20	18			11	6	22
21–25	11			7	5	19
> 25	1			1	–	–

**Fig. 1:**
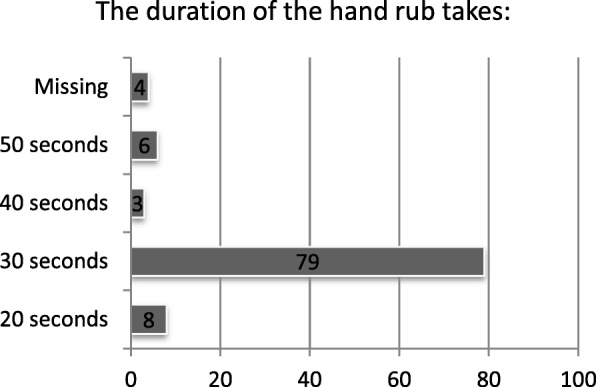
Responses to question about correct duration of hand rub (correct = 30 s)

**Fig. 2:**
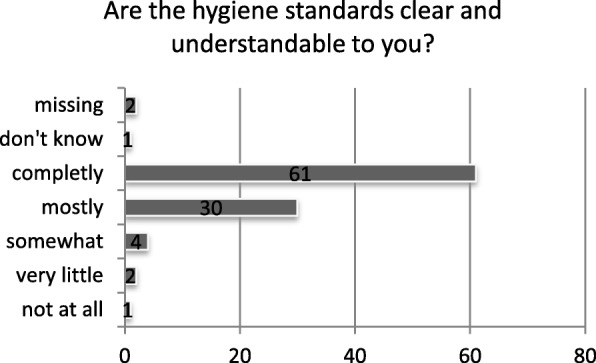
Clarity of hygiene standards

An overview of responses to the staff survey is given in Table 2 as well as in Figs. 1 and 2.

Table 2 presents mean percentages and 95% confidence intervals for the complete sample, nursing aides and registered nurses. Based on Fisher’s exact test, only two items demonstrated a statistically significant difference between professional groups.

**Table 2:** Descriptive survey findings from registered nurses’ and nursing aides’ knowledge and perceived behaviour concerning hand hygiene and infection preventions

	Percent of “Always” or “Often” (Group means and 95% confidence intervals)					
	Complete sample (N = 165)		Nursing Aides/ Students (*n* = 57)		Registered Nurses (*n* = 80)	
1. What knowledge do nurses have concerning hand hygiene?						
Could wearing gloves substitute a hand rub?	6.1%	±3.7%	3.8%	±5.3%	10.0%	±6.6%
Should you rub your hands after taking off gloves?	81.8%	±5.9%	86.5%	±9.4%	81.3%	±8.6%
Do you ask the registered nurse/geriatric nurse questions concerning hygiene?	46.1%	±7.6%	63.5% ^a^	±13.2%	30.0% ^a^	±10.1%
Do you ask the nursing aide/nursing assistant questions concerning hygiene?	6.7%	±3.8%	7.7%	±7.3%	3.8%	±4.2%
Do you ask the hygiene representative nurse questions concerning hygiene?	29.1%	±7.0%	15.4% ^b^	±9.9%	35.0% ^b^	±10.5%
Do you ask the nursing students questions concerning hygiene?	1.8%	±2.0%	1.9%	±3.8%	1.3%	±2.5%
Do you ask the residential nurse questions concerning hygiene?	32.7%	±7.2%	36.5%	±13.2%	33.8%	±10.4%
Do you ask the director of nursing questions concerning hygiene?	21.8%	±6.3%	13.5%	±9.4%	21.3%	±9.0%
Do you ask the executive director questions concerning hygiene?	9.7%	±4.5%	7.7%	±7.3%	6.3%	±5.3%
Do you ask the general practitioner questions concerning hygiene?	7.9%	±4.1%	1.9%	±3.8%	11.3%	±7.0%
2. Which behaviours do nurses report in relation to hand hygiene?						
Is it possible to disinfect your hands while taking care of a resident?	72.7%	±6.8%	65.4%	±13.1%	75.0%	±9.5%
Do you wear hand or arm jewellery during work?	20.6%	±6.2%	19.2%	±10.8%	26.3%	±9.7%
Does the use of gloves damage your skin?	5.5%	±3.5%	9.6%	±8.1%	3.8%	±4.2%
Do you apply the content of the last hygiene training in your daily work?	83.6%	±5.7%	82.7%	±10.4%	83.8%	±8.1%
3. What do nurses report about their compliance with hygiene standards?						
Do you apply the hygiene standards?	82.4%	±5.8%	82.7%	±10.4%	82.5%	±8.4%
Have you ever not disinfected your hands for personal reasons?	3.6%	±2.9%	7.7%	±7.3%	2.5%	±3.4%
Possible reason: “I had a skin defect.”	1.8%	±2.0%	3.8%	±5.3%	1.3%	±2.5%
Possible reason: “I was under time pressure”	4.8%	±3.3%	3.8%	±5.3%	5.0%	±4.8%
Possible reason: “I didn’t think of it.”	3.6%	±2.9%	1.9%	±3.8%	6.3%	±5.3%
Possible reason: “When my hands are moist with hand rub, I cannot put on the gloves.”	17.0%	±5.7%	15.4%	±9.9%	20.0%	±8.8%
Possible reason: “My skin does not tolerate the hand rub.”	5.5%	±3.5%	11.5%	±8.8%	3.8%	±4.2%
Possible reason: “I wear gloves instead of disinfecting my hands.”	6.7%	±3.8%	7.7%	±7.3%	8.8%	±6.2%
Is adherence to hygiene standards discussed during staff handovers? (For example, in case of existing infectious diseases)	58.8%	±7.5%	65.4%	±13.1%	58.8%	±10.9%
4. What are nurses’ perceptions of organisational structures and processes to improve infection prevention?						
Are suitable gloves always available in your residential area?	93.9%	±3.7%	92.3%	±7.3%	95.0%	±4.8%
Is there always an accessible stock of gloves on your residential area?	95.2%	±3.3%	92.3%	±7.3%	97.5%	±3.4%
Is there an accessible stock- pile of protective clothing (gown, mask, and cap) in your residential area?	79.4%	±6.2%	78.8%	±11.2%	77.5%	±9.2%
Do you remember a situation that prevented you from doing a hand rub for operational reasons?	0.6%	±1.2%	0.0%	±0.0%	1.3%	±2.5%
Yes, because there was no hand rub available on the corridor of the residential area.	3.0%	±2.6%	0.0%	±0.0%	5.0%	±4.8%
Yes, because there was no hand rub on the care cart.	0.6%	±1.2%	0.0%	±0.0%	1.3%	±2.5%
Yes, because there was no hand rub in the work room.	1.2%	±1.7%	0.0%	±0.0%	1.3%	±2.5%
Yes, because there was no hand rub in the community room.	4.2%	±3.1%	1.9%	±3.8%	7.5%	±5.8%
Yes, because there was no hand rub at the nursing station.	1.8%	±2.0%	0.0%	±0.0%	2.5%	±3.4%
Yes, because there was no hand rub in the resident’s room.	19.4%	±6.1%	23.1%	±11.6%	20.0%	±8.8%
5. Do nurses see nursing managers as role models regarding infection prevention?	66.7%	±7.2%	73.1%	±12.2%	63.8%	±10.6%

Note: Fischer’s exact test was used to compare different groups, and only two items resulted in a statistically significant difference between registered nurses and assistant nurses

^a^- *p* < 0.001

^b^- *p* = 0.016

#### Nurses’ knowledge concerning hygiene management and hand hygiene

Correct hand hygiene is the most effective activity to prevent nosocomial infections. Therefore, we asked nurses what the recommended duration of hand rub is. The correct answer of 30 s was known by 79% of the respondents (Fig. 1). When asked if wearing gloves substitutes a hand rub, 68% answered correctly with “never”. 52% of the nurses knew they always have to use hand rub after using gloves. 61% of staff answered that hygiene standards were completely understandable to them (Fig. 2). Finally, 25% of the participants saw licensed nurses as their main contact for questions concerning hygiene issues.

#### Nurses’ self-reported hand hygiene behaviour and compliance with hygiene standards

Concerning their own hygiene behaviour, 56% reported that it was always possible to perform hand hygiene while taking care of residents. 21% of the nurses reported wearing hand and arm jewellery always or often while caring for patients. 41% of respondents reported always applying the learnings from their last hygiene training in their daily work. Concerning their perceived compliance with the hygiene standards, 47% of nurses indicated that they always follow standards. Furthermore, 35% of nurses expressed that in cases of existing infectious diseases, hygiene standards were always discussed during shift handover.

#### Nurses’ perceptions of organisational influences on hand hygiene

Nurses reported that there was always (79%) suitable gloves in the residential area and there was always (67%) an accessible stock of protective clothing. 15% answered that they cannot disinfect their hands during active care because there is no hand rub available in the resident rooms. The nurses perceive their direct supervisor as a role model for compliance with hygiene standards (38% “always”).

### Qualitative strand: results of nursing manager interviews

#### Sample characteristics

All the interviewed nursing managers worked in one of the six participating nursing homes. We invited all nursing managers to participate in the study and had a participation rate of 100%. Interviews lasted an average of 14 min (min. 9; max. 40). Most of the interviewed nursing managers were female (89%) and between 50 and 59 years old (33%). More than half of the interview participants (55%) worked as unit managers. All interviewees worked day shifts and had worked between 11 and 15 years (37%) in the participating nursing homes.

#### Nursing managers’ perceptions of nurses’ knowledge concerning hand hygiene

Our interviews revealed a broad range of nursing managers’ perceptions of nursing staff knowledge of hand hygiene practices in place to maintain and further this knowledge. For all nursing homes, it was highlighted that nurses have access to the nursing and hygiene standards at all times to independently further their knowledge. *“First of all, we’ve got a binder with hygiene standards. It is also available in the residential areas, where staff can check things in case of uncertainty. If the material isn’t helping, I’ve got the hygiene representative to back me up, who’s in contact with sources outside this house, where additional info can be obtained.” *(quote 1, Interview partner (IP)1). Nursing managers stated that the terminology used in the hygiene standards was easy to understand and that standards were clearly structured. One nursing home manager had responded to the fact that many nurses are non-native speakers by ensuring access to relevant information in native languages for staff and by providing collegial support for learning about hygiene standards. *“There are different approaches to ensure that most nurses understand the standards. One opportunity is to engage nurses. Our hygiene standards are created by staff members and individual training is carried out while nursing. (…), if residents come back from the hospital with an (…) infection, we always talk about what’s important in the handover, (…), and include the hygiene standards.”* (quote 6, IP15). Another nursing manager also discussed this. *“You also notice that some employees ask questions that need to be explained in more detail. Likewise, for foreign employees, you sometimes need to have a more specific conversation. But, oh well, that’s what we’re here for.”* (quote 2, IP2). Nursing managers also described that in nursing homes, similar approaches were offered to help nurses improve their knowledge.* “I also get support from the practice instructor (educational role) who is very ambitious for everything to run smoothly, just as provided in the hygiene standards.”* (quote 3, IP6).

Another factor mentioned as influencing nurses’ knowledge was text comprehension; particularly concerning unfamiliar terminology and the transfer of expert knowledge into practice. Several strategies of knowledge transfer were described by interview participants however their expectations concerning this knowledge transfer varied greatly. While one nursing manager indicated that everything was clear from the documentation, another manager stated that the nurses do not understand all the standards.* “I always say what’s written it is reasonable for everyone. There’re also these illustrative pictures.”* And *“Sometimes you need to read the principles three or four times before understanding them. Many things are written in percentages, this is not clear to some people. For them, it was learned once at school and was then ticked off.”* (quote 4,IP3 and quote 5, IP5).

#### Perceptions of impacts on nurses’ hygiene training

In one nursing home, proactive planning and employee-oriented alignment of training were referred to as a well-functioning management system (quote 2, IP2). This notion was supported by another nursing manager who expressed a need for hygiene standards to be communicated frequently and actively practiced. *“It still is the case, that we are a little blind, the standards are there, you could become better and say:Hey, look it could be even better!* *It [standards?] often goes down in daily routine. Honestly, I’m that way too sometimes.”* (quote 15, IP9). One interviewee explained that the main hygiene management strategy was to empower employees (quote 6, IP15).

In this nursing home, the knowledge and implementation of hygiene standards were also part of annual agreed targets with nurses who can make suggestions during appraisal regarding specific areas they would like to be trained in that year.

While it was described as common practice to motivate nurses to independently actively close knowledge gaps concerning hand hygiene, interviewees, however, were not always confident that the relevant information was actively sought often enough in cases of uncertainty or that questions were openly asked to clarify any hygiene issues. Some nursing managers even expressed doubts concerning the basic requirement of reading hygiene standards. *“I don’t think anyone from this house has read the hygiene standards. I’m firmly convinced of that. I reckon everyone has signed off on the standards but no one has read them. And I do give them time (for it), but they don’t do it.”* And *“To be honest, I don’t think that non-registered nurses have even read them (hygiene standards).”* (quote 7, IP5 and quote 8, IP4).

All nursing managers expressed their belief that the standards need to be repeated regularly through staff training otherwise they will be forgotten. In one nursing home, the managers highlighted their long-term task of ensuring relevant knowledge is acquired and nurses are applying correct behaviours. They stressed that standards need to be discussed individually as well as collectively.

However, keeping up to date and obtaining support concerning hand hygiene was described as challenging. For example, one manager expressed her frustration when aiming to obtain additional information. *“Sometimes I ask the nursing home manager, but she doesn’t always know everything in detail. I google more often. I research at home, for example for multi-resistant pathogens and often the GPs don’t know what to do, either.”* (quote 9, IP5).

#### Nursing managers’ perceptions of nurses’ hand hygiene behaviours

When nursing managers described nurses’ hand hygiene behaviour they often discussed the availability and use of hand rub during the nursing care of residents. Some interviewees argued against a permanently available hand rub inside resident rooms and bathrooms while nursing. Their reasoning reflected the risk that a cognitively impaired resident might consume the toxic alcohol-based hand rub. The consequence of having to leave a resident’s bathroom frequently for hand rubs was described as unsatisfactory by one residential nurse. *“Most staff wear their wedding rings during care. I (…) try to give them various short internal training. A while ago, I asked the director of nursing for a written guideline about artificial nails and jewellery and she prepared it. But after a little while, some nurses asked me:* *Why can’t we wear our nails like the colleagues on the other units?* *I don’t know why it was so inconsistent! …and since then it has been a constant topic and caused much disagreement in my team.”* (quote 14, IP24). The danger for residents from transmitted pathogens was frequently described as being lower than the risks from drinking denatured hand rub. However, this risk assessment was different when describing care for a resident with an infection. *“They have to go out (to the hallway). To the care trolley, yes. (…) But, in special resident rooms, we have it. (…) In case of infection there is a dispenser in the room.”* (quote 10, IP24). In two other nursing homes, single-use or mobile hand rub bottles were available for staff to take into the resident rooms. *“It is possible; nurses have a care cart that can be placed in front of the door. And you can also take the disinfectant inside the resident’s room, as we don’t have fixed dispensers on the carts. We also have little bottles for our coats.”* And *“We have the possibility to put these little bottles in our jackets or aprons. But the staff rarely does this.” *(quote 11, IP5 and quote 12, IP1). Nevertheless, this example demonstrates that it is not sufficient to simply provide hand hygiene equipment without staff training and guidance.

#### Nursing managers’ perceptions of nurses’ compliance with hygiene standards

The interviewees described consistent leadership and decision-making, the adoption and awareness of role modelling, and empowerment of staff by nursing managers all had an influence on staff compliance with hygiene standards. Nevertheless, nursing managers also described challenges in achieving this in daily work, as illustrated. *“There are colleagues who wear jewellery. They are repeatedly made aware of it not being okay. We have got very clear guidelines. They also know this but they think they can always cheat their way through. Other colleagues present their hands and fingernails as prescribed in the hygiene standards. Because these nurses know I pay attention to it.”* (quote 13, IP8).

The interviews also highlighted the inconsistency in leadership in single units compared to the leadership of the whole nursing home. *“Most staff wears their wedding rings during care. I (…) try to give them various short internal training. A while ago, I asked the director of nursing for a written guideline about artificial nails and jewellery and she prepared it. But after a little while, some nurses asked me:* *Why can’t we wear our nails like the colleagues on the other units?* *I don’t know why it was so inconsistent! …and since then it has been a constant topic and caused much disagreement in my team.”* (quote 14, IP24).

Nursing managers indicated that the verification of compliance is impossible for most areas of care because the care takes place in closed resident rooms. Nevertheless, when care observations were conducted, they frequently noted that hygiene standards were not adhered to. One interviewee reported regularly carrying out inspections and participating in team meetings of all residential areas to verify compliance to hygiene standards. Another interviewee also highlighted the importance of strong, attentive leadership. During regular inspections, she verifies compliance and alerts staff in cases of deviations. Repeated orientation towards the standards within the daily work routine was described as being challenging. *“It still is the case, that we are a little blind, the standards are there, you could become better and say:* *Hey, look it could be even better!* *It [standards?] often goes down in daily routine. Honestly, I’m that way too sometimes.” *And *“Sometimes I observe that the compliance to standards, for example during the catheter change was not completely adhered to.”* (quote 15, IP9 and quote 16, IP14).

#### Nursing managers’ perceptions of organisational factors facilitating or hindering hand hygiene

The challenge of balancing the competing goals of implementing hygiene measures whilst simultaneously preserving social care and a homelike environment was frequently highlighted in the interviews. The assessment of hygiene management in the nursing home was often compared with one’s own home environment and appeared to frame the interpretation of infection prevention in nursing homes. *“Maybe even too much here and there. Because in the nursing home almost everything should be like at home.”* (quote 17, IP17). The lack of clear conceptualisation of the nursing home as a healthcare facility as hindering a consistent organisational approach to hygiene management was also evident in the variable use of disinfecting agents described by nursing managers. IP4: *“Handrub during caring? We have a care trolley always in front of the door, so that you can grab things on the side.”* *Interviewer: “And what happens while caring for residents with infections?”* IP4:* “Then we have special sets in front of the room.”* And *“After that, the General Practitioner will be contacted. Then we prepare the room, a single room, we do have two alternative rooms here.”* (quote 18, IP4 and quote 19, IP1).

Generally, nursing managers reported a change in infection prevention practices and organisational procedures supporting these behaviours in cases of infection. For example, all nursing managers reported that there was always a sufficient supply of gloves and protective clothing available in residential areas. This was ensured by weekly orders. In most facilities, central “Pandemic boxes” containing additional protective material were available to manage pandemic outbreaks or unforeseen infectious diseases. In the case of newly detected infectious diseases, some nursing homes had the capability for resident separation (quote 19, IP1).

#### Nursing managers’ reflections on their function as role models

Nursing managers were partially conscious of being seen as a role model by the nurses. While some pointed out that leading by example requires discipline, some also realised, during the interview, that their role modelling was not as consistent as it should be. *“Although I ask myself now, am I a good role model, if I wear rings myself? (…) I also like to wear the watch on my wrist, but one also tries to discipline oneself and say: I take it (the watch) off now.”* (quote 20, IP1). Interviews also revealed a lack of nursing managers’ self-reflection even for hygiene topics covered in the recurrent training. “*I don’t wear jewellery. But, I wear the wedding ring, yes!* (...) (Laughs) *I never thought about it.* (...) *Perhaps because nothing has happened until now or perhaps because I don’t know about it. I can’t give you an answer to this. But, sure, we repeatedly have training on that topic. You shouldn’t do it. That’s true!” *(quote 21, IP13).

### Triangulation

#### Convergent results

When triangulating data sources, data converged around similar themes expressed by nurses and nursing managers. Both groups shared the perception that hand hygiene and infection prevention are important themes in daily work and recurring education with annual, mandatory hygiene training help to keep knowledge current. We also found shared views on the availability of hygiene equipment, hygiene standards and organisational procedures supporting a need for better understanding of infection prevention practices.

#### Complementary results

It was relevant for nurses and managers that they themselves, their direct supervisors, and the licensed nurses comply with hygiene standards. Thus, most participants reported that during work they orient themselves towards what they learned in hygiene training courses. At the same time, however, referring to their own behaviour, some participants described wearing artificial nails or jewellery on their hands and arms. This inconsistency was often not noticed by study participants. Nursing managers also showed a lack of self-reflection concerning their function as role models. While they highlight the importance of hygiene management in the nursing home, they orient their behaviour towards their own conceptualisations of infection risks and personal attitudes instead of their organisation’s hygiene standards.

#### Divergent results

We found divergent views of nurses and nursing managers concerning knowledge, perceived behaviour and perceived attitudes concerning hand hygiene. While most nurses gave correct answers to questions about hygiene practices such as the duration of hand rub, nursing managers were frequently in doubt about the level of understanding among staff. Furthermore, while nursing managers perceived nurses’ behaviour as adhering to standards most of the time there were pronounced gaps where organisational procedures such as not allowing alcohol disinfectant in resident rooms and bathrooms hindered hand hygiene. The risk of poisoning a resident with disinfectant was perceived as more real than the possibility of nosocomial infection. However, this reasoning was abandoned when a resident was known to have an infection since under those circumstances the disinfectant would be used and deposited in the resident room.

## Discussion

Due to the explosive nature of antimicrobial resistance on the health of the world’s population, the Secretary-General of the United Nations makes it very clear that there is no time to wait for the strengthening of infection prevention in health facilities, as it is central to minimising disease transmission and the incidence and transmission of human disease. To address the unresolved and increasingly global problem of multi- resistant pathogens, hand hygiene in nursing homes is an important topic for study [1–3, 37–39].

In our study, we aimed to improve understanding of the individual and organisational factors relating to compliance with infection prevention management perceived by nursing staff and nursing managers, with the focus on hand hygiene in nursing homes by applying a mixed-methods approach. We collected survey data on nurses’ knowledge, behaviour, and compliance regarding hand hygiene as well as interview data on nurse managers’ perspectives of organisational influence on infection prevention, to explore multiple perspectives in relation to our research questions. Applying a concurrent triangulation approach, we integrated the main results from the staff survey with nursing manager interviews. Those data described their multiple perspectives concerning relevant knowledge, behaviour, compliance and role modelling and were analysed to identify and clarify parallels and discrepancies in the views expressed at the staff and nursing management level.

Since nurses described their hand hygiene behaviour as being influenced through role modelling from nursing managers, the attitudes and resulting management decisions and behaviours of nursing managers might have an impact on the compliance of staff [40, 41]. However, nursing managers who did not thoroughly reflect on their role modelling behaviour described several inconsistencies in their reasoning and hand hygiene compliance due to personal preferences sometimes linked with outdated knowledge. Educational interventions should specifically address this topic to support nursing managers to act more responsibly and consistently as role models within their organisation.

In our results, we found two statistically significant differences between registered nurses and nursing aides/students regarding the importance of hand hygiene. The registered nurses would ask their peer’s hand hygiene questions whereas the nursing aides/students would also ask the registered nurses hygiene related queries, rather than asking their peers. This is a positive sign from a patient safety perspective and unusual compared to the results of similar studies [42, 43]. To sum up, in the current study, the sample does not show any significance on most questions, which is interesting, because the knowledge, attitude, and behaviour on the subject of hand hygiene are similar, despite their different professional qualifications.

Our results indicated one in five nurses had correct knowledge of practical implementation of the hygiene training contents. The 30 s duration time of hand rub was answered correctly by 79%. In contrast, Aiello et al. found that only 40% knew the correct duration time of hand rubbing [9]. The fact that wearing gloves is not a sufficient substitute for a handrub was known by two out of thee of our respondents. This could mean that 33% of the staff did not disinfect their hands after removing the gloves. Application of this knowledge in nurses’ practical work has been found to be related to recurrent hygiene training [43]. However, there were uncertainties expressed by nurses concerning more specific knowledge, for example, in handling cases with multi-resistant pathogens. Similar uncertainties and a corresponding request for special infection prevention training of nurses by nursing managers have also been shown in other studies [17].

Nursing managers shared nurses’ insecurities regarding specific knowledge and were concerned about non-compliant hand hygiene behaviour and cross-infections. Tailored training and repeated guidance are required to improve safe hand hygiene behaviour [11, 28, 44]. In addition, in acute cases, nursing managers described taking a more active role by attending shift handovers and discussing contents of the relevant hygiene standards with the nurses to raise awareness and ensure compliance [42].

Our findings on the availability of protective material showed that it is not problematic to organise and wear protective clothing while nursing. Similar studies reveal comparable results [45]. In some of the participating nursing homes in this study, this was not the case due to organisational risk trade-off decisions that put less emphasis on the potential risk due to invisible pathogens than on the possibility of poisoning residents with alcohol-based hand rub. This example highlighted that continuous risk assessment of cross-infections in long-term care was often not a conscious priority for nursing managers. This imbalance was also fuelled by the conceptualisation of a nursing home as a home-like environment rather than a health care facility. This appeared to impact nursing managers’ decisions and thus organisational policies concerning hand hygiene management and infection prevention and to influence their risk recognition [9].

Interestingly, nurses, as well as nursing managers, reported a shift in hand hygiene practices when residents were diagnosed with a multi-resistant infection. The risks of infection were then prioritised over potential poisoning or harm to the homelike atmosphere. This shift is in line with other research stating that confirmed infection of a resident, for example after a hospitalisation, brings infection prevention into focus [9]. Specifically, nursing managers described that in this event, disinfectants and protective clothing were stored in resident rooms, or in the entrance area and could more easily be used while nursing. This focused behaviour has also been described in hospital settings [43]. Our findings are consistent with previous work by Russell et al., who found similar results with nurses regarding the knowledge nurses have concerning hand hygiene, compliance and attitudes towards infection control measures [45]. In general, many of our findings support the existing literature [45].

Kingston et al. reported skin sensitivity (17% of cases) and skin damage (13% of the cases) associated with hand disinfectants, which may have resulted in the poor acceptance of the hand rubbing by the users [41]. In our study, only 5.5% of the respondents reported intolerance, skin damages and suffering as possible reasons for not using the hand rub, indicating that skin problems, though still prevalent, may not be the major obstacle for use of the hand disinfectants.

We also identified some barriers to hand hygiene behaviour. While staff was motivated to apply the contents of hand hygiene training in practice, their actual compliance appeared to be strongly impacted by the direct availability of hand hygiene equipment while providing nursing care in resident rooms. Other studies also highlight that staff compliance depends on the direct access of hand rub while nursing in resident rooms and bathrooms [9].

With our results, we hope to illustrate the multiple perspectives of healthcare providers that need to be considered when striving for a real-life, contextual understanding of the challenges of hand hygiene management and infection prevention in this field. Our findings contribute to a more comprehensive and nuanced understanding of applied hand hygiene and infection prevention in complex care systems by identifying the role of organisational factors in facilitating or hindering the implementation and management of effective infection prevention in nursing homes [46, 47].

### Limitations

This study was conducted in six nursing homes with different care levels and a minimum of 80 residents per nursing home. While specific requirements concerning infection prevention may differ across national contexts we believe that the organisational influences identified in our sample may well be relevant to other countries. Also, as with all voluntary studies, there may be a selection bias with nurses interested in infection prevention is more likely to participate. Thus, our data may underestimate the prevalence of the phenomena described here. Further, the sample of nursing managers in the qualitative strand of this study may be considered rather small, thus limiting the generalisability of our findings. It should be noted that a sample of 27 participants is not unusually small for an interview study and that there was a natural limitation to the pool of potential participants in managerial roles in the six participating nursing homes. Because interviews were conducted until data saturation was observed, we believe our findings represent the situation in the participating nursing homes sufficiently well and serve as a good foundation for future studies exploring nursing leadership in infection prevention in more detail and with a larger sample. Finally, within the scope of this study, we were unable to obtain additional qualitative information from nurses. This should be considered in future studies to allow for even richer descriptions.

## Conclusion

In summary, our study shows that isolated interventions aimed at improved hand hygiene in nursing homes will demonstrate little effect if not supported by a shared attitude by nurses and nursing managers that hold hygiene management as a priority for resident safety. To raise awareness and facilitate compliant hand hygiene behaviour will require the development of a safety culture along with a shift in nurses’ conceptualisation of nursing homes as healthcare settings with high infection risks. In order to minimise the risk of cross-infection among residents, the nursing managers and the staff should be guided by the WHO recommendations for nursing homes [37] and the national “Action-Clean-Hands” initiative (http://www.aktion-sauberehaende.de). Nursing managers play a key role in facilitating this process in a leadership role but also as role models [9, 28, 40, 48].

## Data Availability

All datasets of study participants are stored according to the data protection requirements in the archive of the University Hospital Bonn, Germany. The datasets generated and analysed during the study and are not publicity available due to terms of written informed consent to which the participants agreed but are available from the corresponding author on reasonable request.

## Abbreviations

IP: Interview Partner
WHO: World Health Organization

## References

[1] World Health Organization, Editor. Worldwide country situation analysis: response to antimicrobial resistance. 1st ed. Geneva; 2015.

[2] Wischnewski N., Mielke M., Wendt C. (2011). Healthcare-associated infections in long-term care facilities (HALT). Bundesgesundheitsblatt - Gesundheitsforschung - Gesundheitsschutz.

[3] Tacconelli Evelina, Pezzani Maria Diletta (2019). Public health burden of antimicrobial resistance in Europe. The Lancet Infectious Diseases.

[4] Allegranzi B, Pittet D (2009). Role of hand hygiene in healthcare-associated infection prevention: proceedings of the lancet conference on healthcare-associated infections. J Hosp Infect.

[5] van den Dool C, Haenen A, Leenstra T, Wallinga J (2016). The role of nursing homes in the spread of antimicrobial resistance over the healthcare network. Infect Control Hosp Epidemiol.

[6] Hughes C, Smith M, Tunney M, Bradley MC. Infection control strategies for preventing the transmission of methicillin-resistant *Staphylococcus aureus* (MRSA) in nursing homes for older people. The Cochrane database of systematic reviews. 2011;12:CD006354. 10.1002/14651858.CD006354.pub3. 10.1002/14651858.CD006354.pub3 22161402

[7] Pittet Didier, Allegranzi Benedetta, Boyce John (2009). The World Health Organization Guidelines on Hand Hygiene in Health Care and Their Consensus Recommendations. Infection Control & Hospital Epidemiology.

[8] Baldwin NS, Gilpin DF, Tunney MM, Kearney MP, Crymble L, Cardwell C, Hughes CM (2010). Cluster randomised controlled trial of an infection control education and training intervention programme focusing on methicillin-resistant Staphylococcus aureus in nursing homes for older people. J Hosp Infect..

[9] Aiello AE, Malinis M, Knapp JK, Mody L (2009). The influence of knowledge, perceptions, and beliefs, on hand hygiene practices in nursing homes. Am J Infect Control.

[10] Huang T-T, Wu S-C (2008). Evaluation of a training programme on knowledge and compliance of nurse assistants' hand hygiene in nursing homes. J Hosp Infect..

[11] Yeung WK, Tam WSW, Wong TW (2011). Clustered randomized controlled trial of a hand hygiene intervention involving pocket-sized containers of alcohol-based hand rub for the control of infections in long-term care facilities. Infect Control Hosp Epidemiol.

[12] Ho Mei-lin, Seto Wing-hong, Wong Lai-chin, Wong Tin-yau (2012). Effectiveness of Multifaceted Hand Hygiene Interventions in Long-Term Care Facilities in Hong Kong: A Cluster-Randomized Controlled Trial. Infection Control & Hospital Epidemiology.

[13] Smith PW, Bennett G, Bradley S, Drinka P, Lautenbach E, Marx J (2008). SHEA/APIC guideline: infection prevention and control in the long-term care facility. Am J Infect Control.

[14] Smith A, Carusone SC, Loeb M (2008). Hand hygiene practices of health care workers in long-term care facilities. Am J Infect Control.

[15] Holton G. Robert K. Merton, 4 July 1910–23 February 2003. Proc Am Philos Soc 2004;148:506–17. 15818878

[16] Huis A, Schoonhoven L, Grol R, Donders R, Hulscher M, van Achterberg T (2013). Impact of a team and leaders-directed strategy to improve nurses’ adherence to hand hygiene guidelines: a cluster randomised trial. Int J Nurs Stud.

[17] Schneider J, Moromisato D, Zemetra B, Rizzi-Wagner L, Rivero N, Mason W (2009). Hand hygiene adherence is influenced by the behavior of role models. Pediatr Crit Care Med.

[18] Lankford MG, Zembower TR, Trick WE, Hacek DM, Noskin GA, Peterson LR (2003). Influence of role models and hospital design on hand hygiene of healthcare workers. Emerging Infect Dis.

[19] Gesundheitsschutz AG (2005). Infection prevention in Nursing Homes: Empfehlung der Kommission für Krankenhaushygiene und Infektionsprävention beim Robert Koch-Institut (RKI). Bundesgesundheitsbl - Gesundheitsforsch - Gesundheitsschutz.

[20] Gould DJ, Moralejo D, Drey N, Chudleigh JH. Interventions to improve hand hygiene compliance in patient care. Cochrane Database Syst Rev. 2010:CD005186. 10.1002/14651858.CD005186.pub3. 10.1002/14651858.CD005186.pub3 20824842

[21] Bruno A, Bracco F (2016). Promoting safety through well-being: an experience in healthcare. Front Psychol.

[22] Ministerium des Innern des Landes Nordrhein-Westfalen. Wohn- und Teilhabegesetz-Durchführungsverordnung: WTG DVO. Düsseldorf; 23. Oktober 2014.

[23] Rahman AN, Applebaum RA, Schnelle JF, Simmons SF (2012). Translating research into practice in nursing homes: can we close the gap?. Gerontologist..

[24] Alp Emine, Ozturk Ahmet, Guven Muhammed, Celik Ilhami, Doganay Mehmet, Voss Andreas (2011). Importance of structured training programs and good role models in hand hygiene in developing countries. Journal of Infection and Public Health.

[25] Cure L, van Enk R (2015). Effect of hand sanitizer location on hand hygiene compliance. Am J Infect Control.

[26] Baldwin A, Mills J, Birks M, Budden L (2014). Role modeling in undergraduate nursing education: an integrative literature review. Nurse Educ Today.

[27] Perry RNB (2009). Role modeling excellence in clinical nursing practice. Nurse Educ Pract.

[28] Sax H, Uçkay I, Richet H, Allegranzi B, Pittet D (2007). Determinants of good adherence to hand hygiene among healthcare workers who have extensive exposure to hand hygiene campaigns. Infect Control Hosp Epidemiol.

[29] Creswell JW (2009). Research design: qualitative, quantitative, and mixed methods approaches.

[30] Houser J (2012). Nursing research: Reading, using, and creating evidence.

[31] Cherry MG, Brown JM, Bethell GS, Neal T, Shaw NJ (2012). Features of educational interventions that lead to compliance with hand hygiene in healthcare professionals within a hospital care setting. A BEME systematic review: BEME guide no. 22. Med Teach.

[32] Lincoln YS, Guba EG, editors. Naturalistic inquiry. Newbury Park, Calif. [u.a.]: Sage; 1985.

[33] Chiang-Hanisko Lenny, Newman David, Dyess Susan, Piyakong Duangporn, Liehr Patricia (2016). Guidance for using mixed methods design in nursing practice research. Applied Nursing Research.

[34] Dresing T, Pehl T. Praxisbuch Interview, Transkription & Analyse.: Anleitungen und Regelsysteme für qualitativ Forschende. 3rd ed.; 2013.

[35] Archibald Mandy M. (2015). Investigator Triangulation. Journal of Mixed Methods Research.

[36] Bogdan R, Biklen SK (2007). Editors. Qualitative research for education: an introduction to theories and methods. 5th ed. Boston, mass.

[37] World Health Organization (2012). SAVE LIVES Clean Your Hands: A Guide to the Application of the WHO Multimodal Hand Hygiene Improvement Strategy and the “My Five Moments for Hand Hygiene” Αpproach.

[38] Tacconelli E (2009). Screening and isolation for infection control: proceedings of the lancet conference on healthcare-associated infections. J Hosp Infect.

[39] Gould D, Moralejo D, Drey N, Chudleigh J, Taljaard M (2018). Interventions to improve hand hygiene compliance in patient care: reflections on three systematic reviews for the Cochrane collaboration 2007-2017. J Infect Prev.

[40] Nicol PW, Watkins RE, Donovan RJ, Wynaden D, Cadwallader H (2009). The power of vivid experience in hand hygiene compliance. J Hosp Infect.

[41] Kingston LM, Slevin BL, O'Connell NH, Dunne CP (2017). Hand hygiene: attitudes and practices of nurses, a comparison between 2007 and 2015. Am J Infect Control.

[42] Leape LL, Shore MF, Dienstag JL, Mayer RJ, Edgman-Levitan S, Meyer GS, Healy GB (2012). Perspective: a culture of respect, part 2: creating a culture of respect. Acad Med.

[43] Lancaster G, Kolakowsky-Hayner S, Kovacich J, Greer-Williams N (2015). Interdisciplinary communication and collaboration among physicians, nurses, and unlicensed assistive personnel. J Nurs Scholarsh.

[44] Ho SE, Ho CCK, Hng SH, Liu CY, Jaafar MZ, Lim B. Nurses compliance to hand hygiene practice and knowledge at Klang Valley hospital. 2013. 10.7417/CT.2013.1604 24217826

[45] Russell D, Dowding DW, McDonald MV, Adams V, Rosati RJ, Larson EL, Shang J (2018). Factors for compliance with infection control practices in home healthcare: findings from a survey of nurses' knowledge and attitudes toward infection control. Am J Infect Control.

[46] Stevens S, Hemmings L, White C, Lawler A (2013). Hand hygiene compliance: the elephant in the room. Healthcare infection.

[47] Cohen CC, Herzig CTA, Carter EJ, Pogorzelska-Maziarz M, Larson EL, Stone PW (2014). State focus on health care-associated infection prevention in nursing homes. Am J Infect Control.

[48] Cheung RB, et al. Nursing care and patient outcomes. International evidence NIH Public Press. EnfermClin. 2008;18:35–40. PMID:18218265. 10.1016/s1130-8621(08)70691-0 PMC2856593 18218265

